# Supplementary figures and images for: Influence of Grafting on Rootstock Rhizosphere Microbiome Assembly in Rosa sp. ‘Natal Brier’
Source: Biology (Basel). 2023 Apr 27;12(5):663. doi: 10.3390/biology12050663 (PMC10215458; doi:10.3390/biology12050663)

a) Compartment

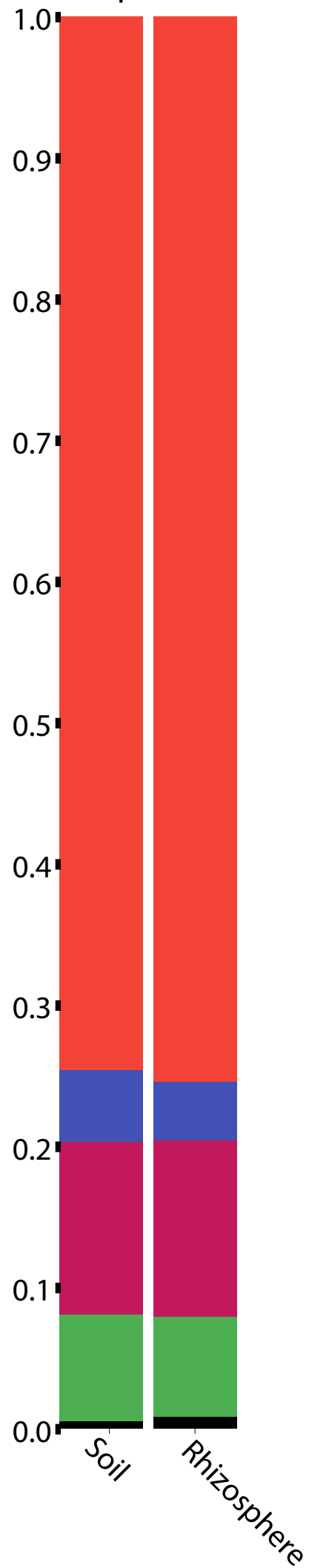

b) Grafting

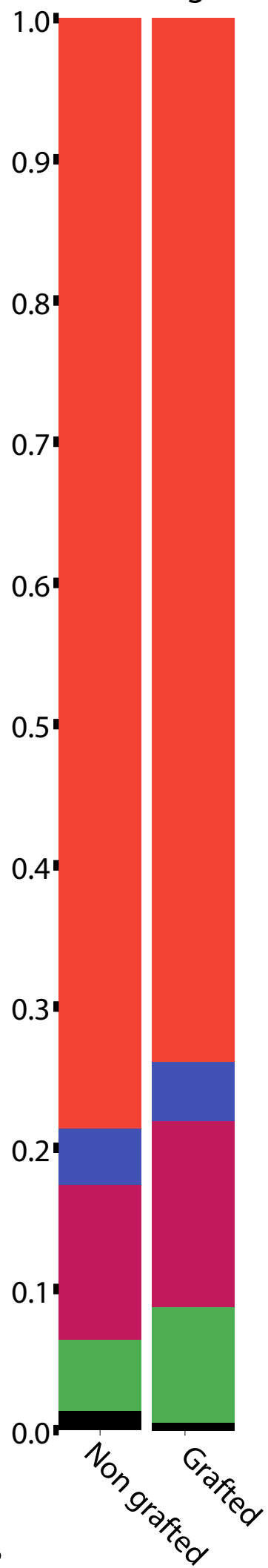

c) Variety

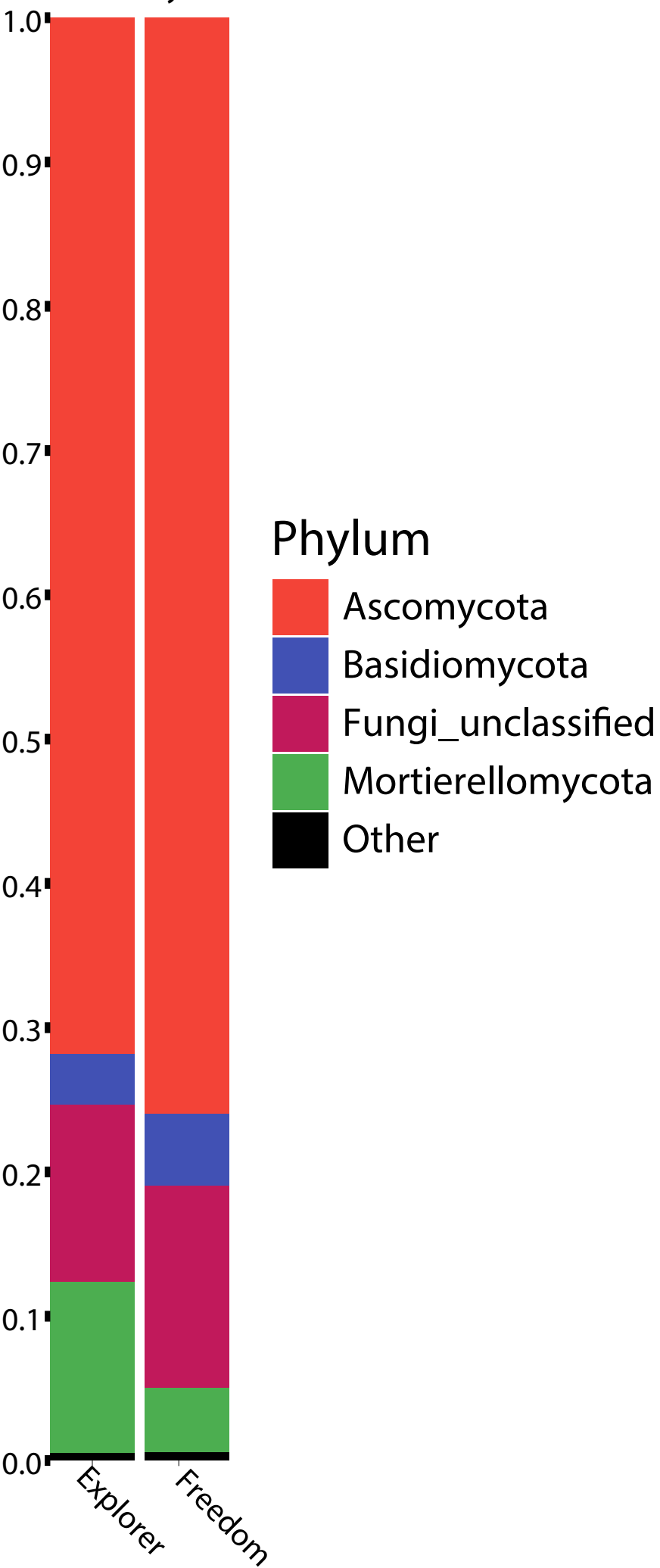

Supplement: Supplementary file 1 [file biology-12-00663-s001.zip › biology-2274859-supplementary-Figure S1.pdf]
